# Communication System Design for an Advanced Metering Infrastructure

**DOI:** 10.3390/s18113734

**Published:** 2018-11-02

**Authors:** Ricardo Siqueira de Carvalho, Pankaj Kumar Sen, Yaswanth Nag Velaga, Lucas Feksa Ramos, Luciane Neves Canha

**Affiliations:** 1 Department of Electrical Engineering, Colorado School of Mines, Golden CO 80401, USA; psen@mines.edu (P.K.S.); yvelaga@mines.edu (Y.N.V.); 2 Department of Electrical Engineering, Federal University of Rondônia, Porto Velho 76801-059, Brazil; feksa@unir.br; 3 Department of Electromechanics and Power Systems, Federal University of Santa Maria, Santa Maria 97105-900, Brazil; lucianecanha@ufsm.br

**Keywords:** communication network, cyber-physical systems, smart grid, advanced metering, power line communication, network simulator 3

## Abstract

This paper primarily deals with the design of an Information and Control Technology (ICT) network for an advanced metering infrastructure (AMI) on the IEEE 34 node radial distribution network. The application is comprised of 330 smart meters deployed in the low voltage system and 33 data concentrators in the medium voltage system. A power line carrier (PLC) communication system design is developed and simulated in Network Simulator 3 (NS-3). The simulation result is validated by comparing the communication network performance with the minimum performance requirements for AMI. The network delay of a single data frame is calculated and compared with the simulation delay. The design methodology proposed in this article may be used for other smart grid applications. The secondary goal is to provide AMI network traffic based on the IEC Std. 61968 and a discussion on whether or not AMI could possibly be a source of big data on the future power grid.

## 1. Introduction

The modern electric grid integrates the existing power system infrastructure with an ICT network allowing for improvements of the system in terms of efficiency, reliability, and flexibility [1,2,3]. The definition of smart grid is adopted from the National Institute of Standards and Technology (NIST) that includes the increased use of digital information and controls technology in the power system [4]. 

Since the amount of data and control in the modern power grid is increasing rapidly as it becomes more mature, the interdependency between the cyber and physical systems increases as well. The ICT network design for smart power applications becomes a more critical task [5]. In the past few years, several surveys have been published addressing the smart grid applications and its communication requirements as well as the ICT technologies [6]. Reference [4] provides a comprehensive overview of the design process of the ICT layer of a smart grid. However, due to the complexity, the design and implementation of the ICT network is still a very challenging task. Different power utilities across the globe have different power network characteristics as well as different geographical features. It is difficult, if not impossible to have a single generalized design procedure that fits all power systems [7]. It is worth mentioning the related AMI work from reference [8] but this research differs from the reference by following the information model provided by IEC Std. 61968.

**Figure 1:**
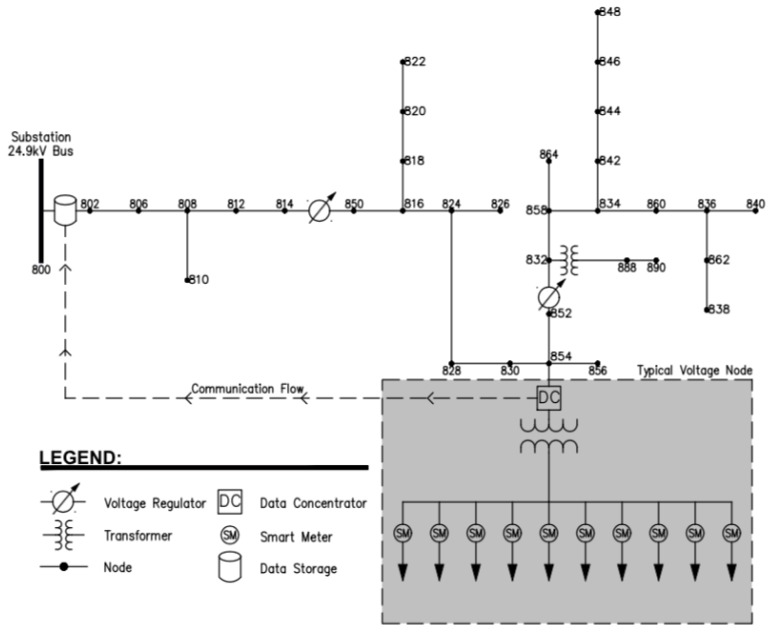
The IEEE 34 bus feeder single line diagram. Modified from [10].

The design process depends on the specific features and requirements. Case studies are valuable for such a design task. This paper performs a case study of an ICT network for an AMI using the Network Simulator 3 (NS-3) software [9] and deployed on the IEEE 34 node radial distribution test network shown in Figure 1 [10]. The distribution feeder is very long and lightly loaded at a 24.9 kV nominal voltage and little over 2000 kVA load. A PLC model library provided in Reference [11] is also utilized.

## 2. Materials and Methods

### 2.1. Communication Network Design Process

**Figure 2:**
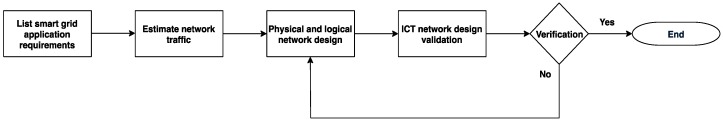
The design methodology flowchart.

A communication network design process similar to the one proposed in [4] is adopted here because its methodology is tailored to power system applications. The flowchart in Figure 2 summarizes the process:

- Smart Grid Application Requirements: The first step is to determine what applications and functionalities are required. Each application has different needs in terms of reliability, security, and performance that collectively comprise the quality of service requirements (QoS). In addition, the communication engineer should also consider physical constraints related to the terrain and device locations as well as the available budget.
- Network Traffic Estimation: The next step is to identify all sources of data traffic. Each source of data may have different sizes of packets, so it is necessary to list all possible data packet sizes and how frequent they are generated. The size of the data traffic depends on the information model of each application. Table 1 summarizes some of the main communication protocols for different smart grid applications. Once all the traffic data types, sizes, and sources are known then the total network traffic estimation is computed to determine the data rate and latency allowed.
- Physical and Logical Network Design: The third step is to determine the physical location of each ICT device. The maximum distance between the ICT devices and what type of physical barriers may exist between them influences the decision for the best communication technologies. The communication technology (or combination of technologies) is determined including the best location forming the topology of the communication network. References [12,13] provide a good summary of the main trade-offs between the commonly used communication technologies for smart grids.
- Network Design Validation: There are two approaches to validate a communication network. (i) First, by running actual experiments with real hardware in the physical location or in a location with similar features as the target location; or (ii) second, by running computer simulations and using communication models that give an approximation of real-world performances. Actual experiments are more accurate, but they are expensive and time-consuming so, usually, validation is done through computer simulations. Some of the communication network simulators mostly used for smart grid applications include the Network Simulator 3 (NS-3), OMNet++, and OPNET [14]. Each simulator has its advantages and disadvantages, but that discussion is beyond the scope of this paper. A thorough discussion on both the communication and power simulators commonly used for smart grid studies has been presented in Reference [1].
- Verification: The final step is to ensure that the network design meets the minimum application requirements (or specification) in terms of QoS. If the design does not meet the needs, then a redesign is done in order to meet the minimum application requirements.

### 2.2. Case Study Details

In the legacy system, the electricity usage was measured locally through an induction-disc electromechanical energy meter. Some utility personnel were responsible for reading and recording those measurements that would be used later for billing purposes. AMI is a bi-directional data communication system that allows for the gathering of energy consumption measurements digitally from electricity customer and sending it to the utility. Additionally, AMI enables demand response (DR) programs, time of day metering, and net metering, among other features [8]. The following subsections expand the design steps for the AMI case study proposed in this paper.

#### 2.2.1. AMI Application Requirements

Each smart grid application has different requirements in terms of reliability, bandwidth, and latency. The minimum requirements for some of the common applications are available in Reference [3]. For the AMI case, the minimum data rate is 56 kbps and the maximum latency is 2 s [3]. However, it is a good practice to design a communication network based on the network traffic estimation that may have more stringent requirements.

The standard communication protocol for AMI, IEC Std. 61968, specifies the information model as well as which network protocols may be used, and it also specifies that the messages between the communication nodes should be done using the extensible markup language (XML) [15]. This network protocol is adopted in this design for the messages between the substation and the smart meters. 

#### 2.2.2. AMI Network Traffic Estimation

**Figure 3:**
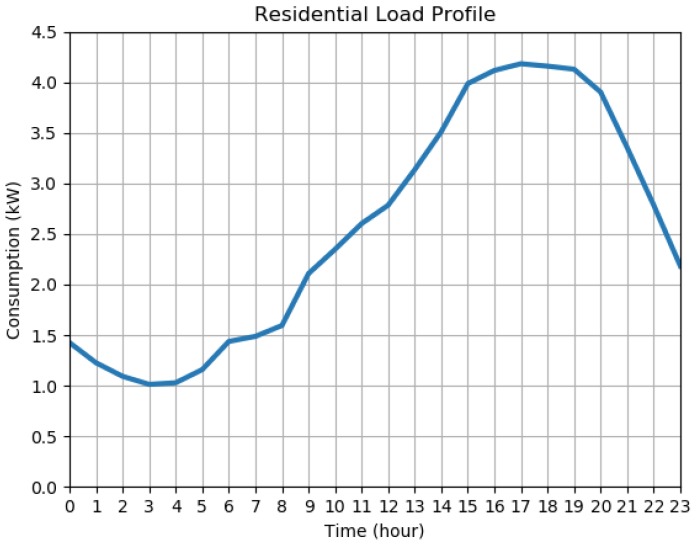
The daily load profile used as the input for all smart meters.

In order to simulate and test the communication scheme, it is necessary to have the load profile for each smart meter. Since the IEEE 34 node feeder is located in Arizona, USA, a typical daily load profile of an average residence from Phoenix, Arizona, available from the OpenEI database [16] is used. For simplicity and proof of concept, the same profile shown in Figure 3 is used for all the smart meters. However, different load profiles for different meters could easily be accommodated. 

**Figure 4:**
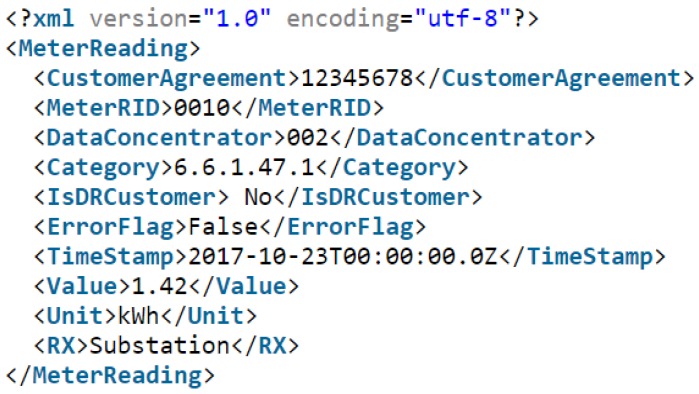
An example of the XML load measurement from a smart meter.

The information model used for structuring the load measurement is based on the IEC Std. 61968 where all the measurement information is structured in one XML file with each type of information categorized by an XML tag as shown in Figure 4 below.

Each measurement from the smart meters follows the above model. The presentation layer of the open systems interconnection (OSI) model is chosen to be UTF-8 that uses one-byte encoding. Every character symbol in the information model adds one byte to the total size of the packet. Every measurement message has 381 characters and, therefore, 381 bytes. The total network traffic estimation can be given by the summation of all data from all data sources:

(1) $$\mathrm{Network}\;\mathrm{Traffic}\;\mathrm{Estimation}=\sum_{k=0}^{n}\left(PS_{k}+OS_{k}\right)\times NDS_{k},$$

where, PS and OS are the payload size and overhead size of a given data source, and NDS is the number of data sources for a given application. It is common for smart grids to have multiple applications and multiple types of data sources. However, this project considered only an AMI application. Table 2 outlines the network traffic estimation for this project that is 128,370 bytes. This is the total amount of data flowing in the communication network during every measurement interval. The interval for AMI according to IEC Std. 61968 is between 15 to 60 min, depending on the utility. In this design, the interval is chosen to be 60 min due to the public dataset used that had hourly granularity.

#### 2.2.3. AMI Communication Network Design—Choosing the ICT Technology

With a network traffic estimation of 128,370 bytes per hour, the majority of technologies available such as PLC, IEEE Std. 802.16 (WiMAX), IEEE Std. 802.11 (Wi-Fi), or fiber optics could easily handle this data rate [12,13]. In this project, each one of the data concentrators is physically located at each transformer and based on the physical topology, the longest section between the transformers is around 11 km [10]. Due to the aforementioned distance, it is possible to eliminate many technology options that are meant for distances shorter than 11 km such as IEEE Std. 802.15.4 and IEEE Std. 802.11 [12,13]. The technology options that have physical range capabilities greater than 11 km area are IEEE Std. 802.16 (WiMAX), Fiber, or narrowband PLC. Among the three technologies, PLC is the one with the lowest cost since it utilizes the existing power line as the communication medium avoiding higher installation costs when compared to fiber and WiMAX [12,13].

The data rate of narrowband PLC ranges from 5 to 500 kbps [17] and it is chosen for this project. The data rate is set to 130 kbps in order to leave room for scalability. The modulation technique choice is binary phase shift keying (BPSK) due to its inherent reliability. The medium access control (MAC) algorithm is carrier sense multiple access (CSMA) with collision avoidance (CSMA/CA). That is, the MAC algorithm is specified by the IEEE Std. P1901.2 which is the Std. for narrowband PLC [17].

#### 2.2.4. AMI Cyber Security

AMI like any other smart grid application devices is exposed to cyber threats. The two major cyber-attacks targeting AMI systems are (a) hackers aiming to gain access to confidential data from customers so they can infer the house scheduled based on the load profile, so one may plan a robbery based on such information; and (b) customers may hack and change the energy usage data to less than the real amount in order to reduce their energy bills as a form of energy theft [18,19]. Thus far, several cyber-security measures for AMI have been proposed in the literature [20], however, since cyber-security is not the focus of this research, those measures were not implemented in this project. Nevertheless, cyber-security is a very important part of the smart grid communication design. 

#### 2.2.5. PLC Network System Modeling

The PLC communication network is modeled using the C++ and the NS-3 simulator. The electrical model for overhead lines, transformers, and other electric devices as well as the channel model is developed in [11]. This is the base for building the physical electric model. As mentioned earlier, 330 smart meters deployed in the low voltage system and each meter is connected to one of the 33 data concentrators located in the high voltage side of each distribution transformers. Each data concentrator gathers the hourly load data from the controller located in the substation and pulls all the measurement data. The information gathered at the substation controller may be used later for both billing purposes and for demand response programs. Each data concentrator is capable of transmitting data at 130 kbps with binary phase shift keying (BPSK) modulation and carrier sense multiple access with collision avoidance (CSMA/CA) as the medium access control protocol in order to make shared medium communication possible.

In this design, the transport and network layers are not used because they would increase the overhead size of the data traffic without adding relevant benefits since the computer network only has 33 data concentrators and one substation controller. The smart meter device models developed here mimic the behavior of real smart meters by sending hourly power measurements whenever the substation sends metering requests. Table 3 summarizes all the simulation parameters for the developed PLC network model.

The main objective of the simulation is to measure the total delay of every single smart meter measurement by using the propagation model and a MAC protocol. In order to count how many measurements are received at the substation and to calculate the signal to noise ratio the PLC receiver modem sensitivity is set to −20 dBm. The communication signal is transmitted from the data concentrators to the substation with the transmission power set to 1 W and it arrives at the receiver with some signal attenuation due to fading over the overhead lines. If the received signal has less than 100 mW power, the receiver is not able to identify the message, therefore, causing a packet drop.

## 3. Results

### 3.1. NS-3 Simulation Results

**Figure 5:**
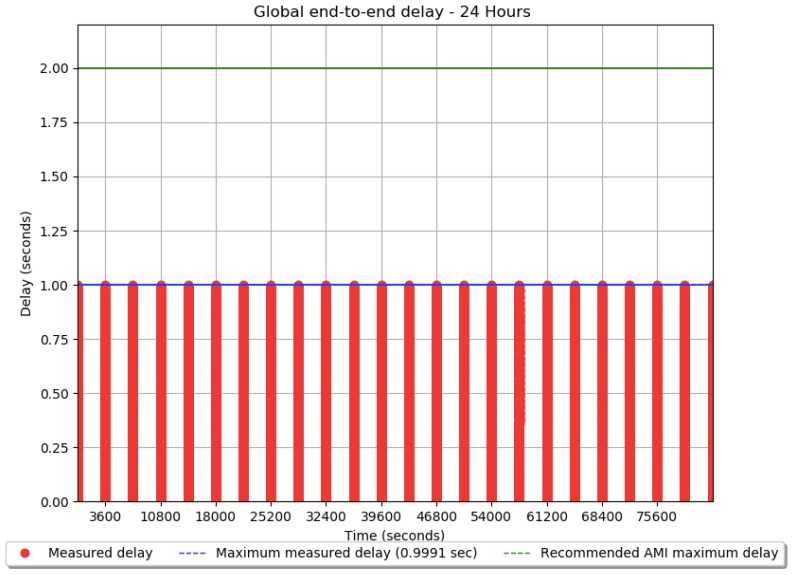
The global end-to-end delay during the daily cycle.

The ICT model is simulated for a period of 24 h. The global end-to-end delay between every smart meter and controller at the substation is measured totaling 7920 measurement packets. Figure 5 depicts the delay results of all packets, both the smart measurement packets as well as additional control messages from the CSMA/CA algorithm used in the MAC sublayer. The total number of messages is over 15,000. The maximum delay is 0.9991 s. All the measurement delays were below 2 s, which is above the recommended communication requirements for AMI.

From the simulation results seen in Figure 5 it is possible to observe that all measurement cycles have the same maximum measured delay of 0.9991 s due to the fact that the number of measurements in every hour is the same and the CSMA/CA algorithm is used so there is no random component to the total end-to-end delay. Additionally, the ICT network is idle during the majority of time throughout the day since all measurements are gathered at the beginning of every hour then there are no messages being transmitted in the ICT network until the following hour. Further, the measurements cycles were scheduled to start on the first second of every hour so there is an equal spacing between the delay “spikes” shown in this aforementioned Figure 5. 

**Figure 6:**
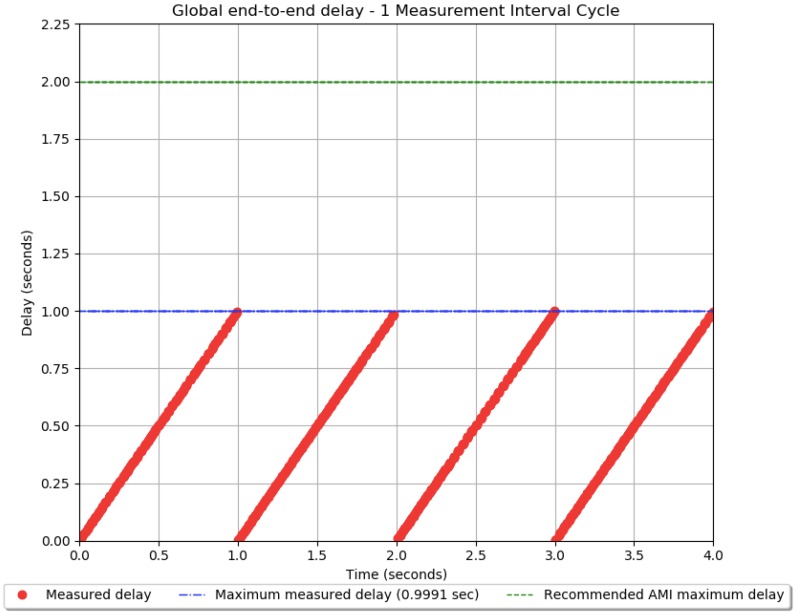
The global end-to-end delay during the one measurement cycle.

The following figure shows more details about the global end-to-end delay in the first measurement cycle that last for less than 4 s (Figure 6). 

In Figure 6 the global end-to-end delay curve has a line shape due to the fact that the CSMA/CA algorithm creates a first come first serve queue of all measurements to be sent through the PLC network since it only allows one communication node to use the shared medium at a given time, then the global delay increases proportionally to the number of messages to be sent at the same time. If CSMA/CD or some other MAC algorithm with collision detection was issued instead, this delay plot would have a stochastic component instead as well as likely packet collisions. 

Another aspect of the delay plot is that there are four lines for one measurement cycle as seen from Figure 6. This is because a time multiplexing scheme is used for reducing the delay where the total 330 smart meters was split into three groups made up of 80 smart meters and one group comprising of 90 smart meters. Besides time multiplexing two other options for reducing the delay in this project are frequency multiplexing and the increase of transmission data rate. Both options may be added later if the number of smart meters increases in the future for the sake of scalability. Additionally, all the 7920 measurement packets were received at the substation node with a 100% availability and the average global end-to-end delay is about 0.5 s. Finally, the minimum measured delay is 0.02393 s. Table 4 summarizes the main simulation results.

### 3.2. Numerical Analysis and Model Validation

In order to validate the NS-3 simulation results, the authors used the end-to-end delay numerical analysis from reference [21]. This can be calculated as

(2) $$\begin{array}{cc} \mathrm{End}\;\mathrm{to}\;\mathrm{end}\;\mathrm{Delay}\;(d)\;= & \;\mathrm{Queuing}\;\mathrm{Delay}\;(d_{queuing})\;+\;\mathrm{Transmission}\;\mathrm{Delay}\;(d_{transmission})\\ & +\;\mathrm{Propagation}\;\mathrm{Delay}\;(d_{propagation}), \end{array}$$

where the Queuing Delay depends on both the network topology and the MAC algorithm (CSMA/CA in this case) and is not trivial to compute. For a single data frame when the channel is free it can be assumed to be zero seconds. The Transmission Delay is given by

(3) $$\;d_{transmission}=\;\frac{Frame\;Lenght}{Channel\;Capacity}\;\;$$

The Propagation Delay depends on the path length and on the propagation velocity that has been assumed to be close to the speed of light (approximately 3 × 10^8^ m/s):

(4) $$d_{propagation}=\;\frac{Path\;Lenght}{Propagation\;Velocity}\;$$

The end-to-end delay of 1 data frame in the link between the node 802 and the substation node (node 800) has been compared with the measured delay from the NS-3 simulation. In this case, since the channel is free there is no Queuing Delay, therefore, $d_{queuing}=0\;s$. The frame size is 389 bytes (3112 bits) and the channel capacity is 130,000 bits/s. The distance between nodes 800 and 802 is 786 m [10] and the propagation velocity is assumed to be the same as the speed of light. Using Equation (2) the calculated End-to-end Delay for this case will be

$$\;d=\;0+\frac{3112\;\mathrm{bits}}{130,000\frac{\mathrm{bits}}{s}}+\frac{786\;m}{3\times 10^{8}\frac{m}{s}}=\;0.02393\;+\;0.0000026,\;d=\;0.0239326\;s$$

which is close to the simulated value of 0.02393 s. From the numerical analysis, it can be noticed that the main component of the delay is the Transmission Delay, in this case.

## 4. Discussion and Conclusions

This paper presents a narrowband PLC communication design and an analysis for an AMI application deploying 330 smart meters along the IEEE 34 bus distribution network. This is modeled in Network Simulator 3 (NS-3) and is compared with the communication network performance requirements for AMI. The network traffic is 285.26 bits per second, which is considered to be very low when compared to state-of-the-art communication technologies. The simulation results show that the proposed communication network meets the minimum requirements and the following lessons are learned:

- Among all communication technologies applicable to modern power systems there is no one “best” technology. Each technology has its pros and cons and the communication engineer should look for the options that meet all the project requirements and the design criteria at minimum cost. Additionally, the ICT design could be a combination of two or more ICT technologies. This is usually the case for smart grids with large geographical areas and/or multiple applications.
- PLC communication has proven to be a cost-effective solution for this AMI application with scope for further scalability without changing the ICT network. The network design steps proposed here are generic and relevant regardless of the different project requirements that may be used for other smart grid applications.
- When there are multiple applications for a smart grid, it is necessary to assign different priorities to different applications. For instance, if an ICT network is being used for both AMI and tele-protection applications then the ICT network should prioritize all messages from tele-protection because it is a more critical application.
- The network traffic of a single smart meter in this study is 0.86 bits per second. When compared to other smart grid applications this traffic is considered very low. For instance, IEEE Std. C37.118.2 for synchrophasor data points that a phasor measurement unit (PMU) with 60 Hz sampling generates 23,040 bits per second which is much higher than a smart meter data rate. Based on those numbers more than 26,000 smart meters would be necessary to generate the same amount of data of a single PMU. Because of this reason, the authors believe that smart metering is not among the applications driving big data in power systems.

## Figures and Tables

**Table 1:** The power system communication protocols [3].

Application	Communication Protocols
Tele-Protection Relays	IEC 60834 or IEEE C37.236
Substation Automation	IEC 61850
Transmission Automation	IEC 60870-5 or DNP3
Synchrophasors	IEEE C37.118.1
AMI	IEC 61968
Demand Response (DR)	IEC 61968
DER Control	IEC 61850

**Table 2:** The communication network traffic estimation.

AMI Network Traffic Estimation	
Payload Size (PS)	381 Bytes
PLC Overhead Size (OS)	8 Bytes
Total Measurement Size	389 Bytes
Number of Smart Meters (NDS)	330 Units
Network Traffic Estimation	128,370 Bytes per hour

**Table 3:** The simulation parameters.

Protocol Layer	Parameter	Value
Application	Packet Size	381 Bytes
	Overhead Size (PLC)	8 Bytes
Presentation	Encoding	UTF-8 (1 Byte/Char.)
Session	Not Using	-
Transport	Not Using	-
Network	Not Using	-
Data Link and MAC	Medium Access Control	CSMA/CA
Physical	Modulation Technique	BPSK
	Chanel Bit Rate	130 kbps
	Transmitted Power	1 Watt
	Receiver Sensitivity	100 mW
	Average Signal to Noise Ratio (SNR)	5 dB

**Table 4:** The simulation results.

Result	Measured	Std. Requirement
Min. Delay	0.02393 s	2 s (max.)
Max. Delay	0.9991 s	2 s (max.)
Average Delay	0.5 s	2 s (max.)
Data Rate	130 kbps	56 kbps
Availability	100%	99.99%
